# GTPLM-GO: Enhancing Protein Function Prediction Through Dual-Branch Graph Transformer and Protein Language Model Fusing Sequence and Local–Global PPI Information

**DOI:** 10.3390/ijms26094088

**Published:** 2025-04-25

**Authors:** Haotian Zhang, Yundong Sun, Yansong Wang, Xiaoling Luo, Yumeng Liu, Bin Chen, Xiaopeng Jin, Dongjie Zhu

**Affiliations:** 1 School of Computer Science and Technology, Harbin Institute of Technology, Weihai 264209, China; zhanght282018@163.com (H.Z.); hitffmy@163.com (Y.S.); yansongwang_hit@stu.hit.edu.cn (Y.W.); bchen@hit.edu.cn (B.C.); 2 Department of Electronic Science and Technology, Harbin Institute of Technology, Harbin 150001, China; 3 College of Computer Science and Software Engineering, Shenzhen University, Shenzhen 518060, China; xiaolingluoo@outlook.com; 4 College of Big Data and Internet, Shenzhen Technology University, Shenzhen 518118, China; liuyumeng@sztu.edu.cn

**Keywords:** protein function prediction, PPI networks, dual-branch graph transformer, graph neural networks, protein language model

## Abstract

Currently, protein–protein interaction (PPI) networks have become an essential data source for protein function prediction. However, methods utilizing graph neural networks (GNNs) face significant challenges in modeling PPI networks. A primary issue is over-smoothing, which occurs when multiple GNN layers are stacked to capture global information. This architectural limitation inherently impairs the integration of local and global information within PPI networks, thereby limiting the accuracy of protein function prediction. To effectively utilize information within PPI networks, we propose GTPLM-GO, a protein function prediction method based on a dual-branch Graph Transformer and protein language model. The dual-branch Graph Transformer achieves the collaborative modeling of local and global information in PPI networks through two branches: a graph neural network and a linear attention-based Transformer encoder. GTPLM-GO integrates local–global PPI information with the functional semantic encoding constructed by the protein language model, overcoming the issue of inadequate information extraction in existing methods. Experimental results demonstrate that GTPLM-GO outperforms advanced network-based and sequence-based methods on PPI network datasets of varying scales.

## 1. Introduction

Proteins are fundamental to biological processes, and accurate resolution of protein functions is crucial for studies such as revealing disease mechanisms and discovering drug targets [1]. Protein functions are standardized by Gene Ontology (GO) [2] through a structured, controlled vocabulary, which involves three aspects: biological process ontology (BPO), molecular function ontology (MFO), and cellular component ontology (CCO). With advancements in sequencing technologies, protein sequence data are increasing rapidly [3]. At present, the number of protein sequences in UniProt has surpassed 250 million [4]. However, due to expensive and time-consuming biochemical experiments, only approximately 0.23% of these sequences have reliable functional annotations [5]. This indicates that biochemical experiments are insufficient to meet the growing demand for protein function annotation. Consequently, it is imperative to develop protein function prediction methods [6,7].

Various data sources can be used for protein function prediction. Sequence data are especially prevalent due to their abundance, which has driven significant technological evolution of sequence-based methods. Classical sequence-based methods, such as Blast [8], PSI-Blast [9], and Blast2GO [10], typically transfer annotations between homologous proteins by sequence similarity comparison [6,11]. The development of deep learning has led researchers to incorporate neural network models for sequence analysis. For instance, DeepGO [12] and DeepGOPlus [13] utilize convolutional neural networks to extract feature information from protein sequences, while TALE [14] is based on Transformer [15], which captures global patterns in sequences. Recent developments have also seen the integration of natural language processing (NLP) techniques, with the introduction of protein language models (pLMs) such as SeqVec [16], ProtTrans [17], and ESM-1b [18]. These models, built upon pre-trained models like ELMo [19] and BERT [20], leverage large-scale unlabeled sequences for pre-training and provide high-quality functional semantic encoding for protein function prediction.

In general, the value of sequence data lies in two aspects: (1) providing vast sequence resources that support data-driven research and (2) offering key biological features, such as conserved structural domains and functional sites, which are essential for analyzing the biochemical properties of proteins. To improve the comprehensiveness and accuracy of protein function prediction, it is essential to examine the functional roles of proteins within biological systems [21,22]. Protein–protein interaction (PPI) networks represent proteins as nodes and characterize their interactions through weighted edges [23]. This network topology can effectively reveal functional collaboration among proteins. Therefore, PPI networks are also crucial for protein function prediction [22,24].

Early studies primarily used unsupervised network embedding methods [25] for PPI network information extraction. For instance, DeepGO [12] constructs a heterogeneous network based on PPI networks and generates embeddings of proteins using the DeepWalk algorithm [26] and skip-gram model [27,28]. deepNF [29] integrates multisource PPI networks, constructs a Positive Pointwise Mutual Information (PPMI) matrix via a random wandering strategy, and learns low-dimensional representations through an autoencoder. Recently, GNNs [30] have proven to be highly effective in biological networks. For example, sAMPpred-GAT [31] uses a Graph Attention Network (GAT) to capture residue-level features and structural information from predicted peptide structures to predict antimicrobial peptides (AMPs). Additionally, in the field of protein function prediction, several studies have also utilized GNNs. DeepGraphGO [32] utilizes a two-layer graph convolutional network (GCN) [33] for feature learning across PPI networks, and it is one of the most advanced network-based methods. HNetGO [34] employs a graph neural network based on attention mechanisms for protein embedding learning. Notably, although existing network-based methods effectively capture local features of PPI networks, the performance is constrained by the message passing mechanism. Excessive stacking of graph network layers can lead to the over-smoothing problem [35,36] and ultimately limit the ability to capture global PPI features.

In PPI networks, proteins are connected through direct interactions. However, even in the absence of direct interactions, proteins may still maintain functional relationships through multihop connections, suggesting that distant proteins can indirectly collaborate in biological processes. Therefore, effective collaborative modeling of local–global features in PPI networks is crucial for further enhancing prediction accuracy. In recent years, Transformer [15] has achieved success across various domains, including computer vision and graph data analysis. Researchers have developed several Graph Transformer models [37,38,39,40]. The global attention mechanism of Transformer enables it to capture long-range dependencies and provides novel approaches for PPI network analysis. For example, CFAGO [41] extracts first-order neighborhood features of proteins via a weighted neighborhood matrix of PPI networks, and enhances its ability to model global information through the Transformer encoder. However, CFAGO’s extraction of local information is limited to its inputs. To date, existing methods have not yet achieved the collaborative modeling of local and global information of PPI networks. Dual-branch architectures have demonstrated their effectiveness in processing different scales or types of information across various fields. For instance, ViLBERT [42] improves multimodal task performance by simultaneously processing visual and linguistic modalities, while Conformer [43] combines a CNN and Transformer to jointly model both local image details and global context. In protein function prediction, Struct2GO [44] verifies the potential of the dual-branch architecture by integrating sequence and structural features. DeepFMB [45] and DeepFMB+ [45] can be considered as multibranch neural network architectures, which enhance protein function prediction performance by integrating sequences, PPIs, and orthology relations. Building on these concepts, constructing a two-branch graph network for protein–protein interaction (PPI) networks to capture both local and global information may be an effective approach to improving prediction accuracy.

In this study, we propose GTPLM-GO, a protein function prediction method that integrates local and global information within PPI networks, as well as sequence data. Specifically, we developed a dual-branch Graph Transformer to achieve the collaborative modeling of local and global information within the PPI network while also incorporating functional semantic encoding of sequences generated by a protein language model, SeqVec [16]. GTPLM-GO significantly enhances protein function prediction performance. This paper makes the following key contributions:

- We propose a novel protein function prediction method, GTPLM-GO, which effectively utilizes the functional semantic encoding of sequences, as well as local and global information of PPI networks. GTPLM-GO enhances protein function prediction accuracy by leveraging the complementarity of this information.
- We develop a dual-branch Graph Transformer that integrates both local and global information from PPI networks. The local information is extracted using a two-layer GCN branch, while the global information is captured through a linear attention-based Transformer encoder. This design effectively mitigates the over-smoothing problem commonly found in traditional graph networks and achieves collaborative modeling of local and global information.
- Experimental results on PPI network datasets of varying scales show that GTPLM-GO outperforms advanced network-based and sequence-based methods. This confirms its ability to extract valuable information from PPI networks and validate information complementation. Furthermore, GTPLM-GO exhibits notable advantages in species-specific protein function prediction, indicating its good generalization capabilities.

## 2. Results

### 2.1. Experimental Setup

GTPLM-GO is trained and evaluated for MF, BP, and CC separately. To optimize the hyperparameters, we used wandb (https://wandb.ai/site) as a hyperparameter search tool to prefer a set of optimal hyperparameters based on the valid set loss values. Subsequently, we apply the trained model to the test set to make predictions and evaluate results. In terms of parameter settings, specifically, the batch size is 1024, the dual-branch Graph Transformer hidden dimension size d=1024, the number of layers in the GCN branch =2, the number of attention layers in the linear attention-based Transformer encoder =1, the number of attention headers =1, and dropout hyperparameters are {0.2,0.3,0.4,0.5}. We use Adam optimizer [46], with learning rate and weight decay values as hyperparameters and the search rates for learning rate in {$1\times 10^{-5},2\times 10^{-5},5\times 10^{-5},1\times 10^{-4},5\times 10^{-4}\}$ and for weight decay in $\left\{1\times 10^{-5},1\times 10^{-6},0\right\}$, respectively. Additionally, we use an early-stopping strategy, limiting the maximum number of training epochs to 1000, and the Patience value is 15, meaning training is stopped early if the valid set loss no longer decreases within 15 epochs. We trained the benchmark methods following the original authors’ implementations. As a network-based method, GTPLM-GO uses only those training samples that appear in both the train set and the PPI network, which is consistent with DeepGraphGO. Other benchmark methods utilize all training samples.

### 2.2. Evaluation Metrics

We used Fmax and AUPR as evaluation metrics, which are widely used to evaluate the performance of protein function prediction [11,32,41,44,47]. AUPR represents the area under the Precision–Recall curve (P-R curve), which is widely used in unbalanced datasets, as well as multilabel classification tasks [11,32]. Fmax is the primary evaluation metric of CAFA [6,7], and can be defined as follows:

(1) $$F_{max}=\underset{\tau \in [0,1]}{max}\left\{\frac{2\times pr(\tau )\times rc(\tau )}{pr(\tau )+rc(\tau )}\right\}$$

where pr(τ) and rc(τ) represent precision and recall computed at the threshold τ, which can be defined as

(2) $$pr\left(\tau \right)=\frac{1}{N(\tau )}\sum_{i=1}^{N(\tau )}\frac{\sum_{j}s(p_{ij}\geq \tau \;and\;f_{j}\in F\left(i\right))}{\sum_{j}s(p_{ij}\geq \tau )}$$

(3) $$rc\left(\tau \right)=\frac{1}{N_{t}\left(\tau \right)}\sum_{i=1}^{N_{t}\left(\tau \right)}\frac{\sum_{j}s(p_{ij}\geq \tau \;and\;f_{j}\in F\left(i\right))}{\sum_{j}s(f_{j}\in F\left(i\right))}$$

where N(τ) denotes the number of proteins with a predicted score of at least one GO term that is not less than the threshold τ, $N_{t}\left(\tau \right)$ denotes the number of proteins in the test set, s(·) is the function used to convert the boolean value “true” to 1 and “false” to 0, $f_{j}$ represent GO term *j*, and F(i) denotes the set of GO terms associated with protein ${prot}_{i}$.

### 2.3. Comparison with Advanced Methods on PPI Networks of Varying Scales

As shown in Table 1, we use Fmax and AUPR as metrics to compare GTPLM-GO with six benchmark methods on the benchmark dataset. For details on benchmark methods, see Supplementary Materials. The benchmark dataset contains a large-scale PPI network constructed based on a multispecies strategy. Experimental results demonstrate that GTPLM-GO surpasses all competing methods in both Fmax and AUPR.

Specifically, GTPLM-GO outperforms the advanced network-based method DeepGraphGO in all metrics. In terms of Fmax, GTPLM-GO achieves performance improvements of 2.1%, 2.9%, and 1.3% in BPO, MFO, and CCO, respectively, compared with DeepGraphGO. For AUPR, the improvements are 11.3%, 4.4%, and 4.3%, respectively. Results show that GTPLM-GO has excellent performance on large-scale datasets. Furthermore, both GTPLM-GO and DeepGraphGO, as models that incorporate sequence and PPI network, outperform the four sequence-based methods. Compared with the best results of the sequence-based methods across six metrics, GTPLM-GO improves Fmax by 15.2% (BPO, DeepGOPlus), 3.9% (MFO, LR-InterPro), and 4.3% (CCO, DeepGOPlus). For AUPR, GTPLM-GO shows improvements of 50.0% ( BPO, LR-InterPro), 7.0% (MFO, LR-InterPro), and 42.5% (CCO, LR-InterPro). These results suggest that GTPLM-GO enhances prediction accuracy by combining the functional semantic coding extracted from the protein language model with local and global information within the PPI network.

Notably, the performance of all methods on BPO is significantly lower than that of MFO and CCO. This discrepancy may stem from the complexity of biological processes involved in BPO, which typically require interactions between multiple proteins and network regulation. Additionally, BPO contains a large number of GO terms, which increases the prediction difficulty. However, the performance improvement of GTPLM-GO on BPO is significant, achieving an 11.3% increase in AUPR. This is probably because proteins connected within PPI networks through both direct (physical) and indirect (functional) interactions are typically involved in common biological processes. GTPLM-GO’s dual-branch Graph Transformer efficiently models the PPI network by incorporating both local and global information, resulting in enhanced performance.

**Figure 1:**
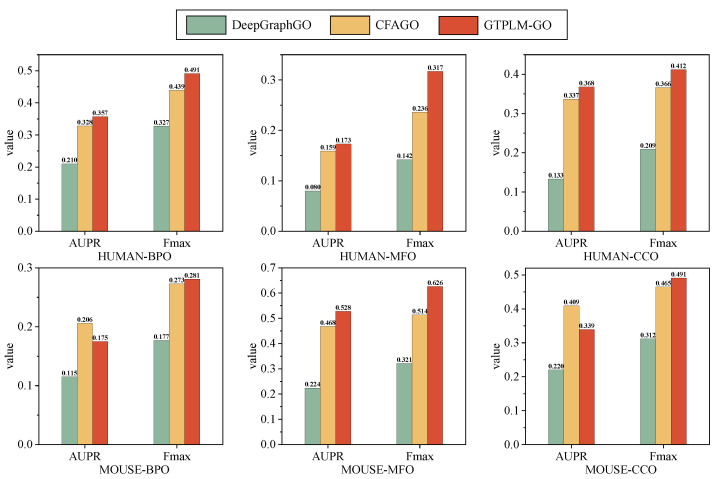
Performance comparison of GTPLM-GO with advanced methods on the CFAGO dataset.

To further evaluate the performance and adaptability of GTPLM-GO on PPI networks of varying scales, we employed the dataset proposed by CFAGO [41]. Details about CFAGO and its proposed dataset can be found in the Supplementary Materials (Table S1). The CFAGO dataset contains two subsets: HUMAN and MOUSE, each containing a small-scale, single-species PPI network. We retrained GTPLM-GO and compared its performance against DeepGraphGO and CFAGO. As shown in Figure 1, GTPLM-GO consistently maintains its superior performance. Specifically, on the human dataset, GTPLM-GO surpasses DeepGraphGO (0.327, 0.142, 0.209) and CFAGO (0.439, 0.236, 0.366), achieving an Fmax of 0.494, 0.329, and 0.412 for BPO, MFO, and CCO, respectively. Similarly, on the mouse dataset, GTPLM-GO attains an Fmax of 0.284, 0.626, and 0.495 for BPO, MFO, and CCO, respectively, outperforming DeepGraphGO (0.177, 0.321, 0.312) and CFAGO (0.273, 0.514, 0.465). These results indicate that GTPLM-GO excels even in small-scale PPI networks, highlighting its strong adaptability.

### 2.4. Generalization on Proteins Within the PPI Network

The protein samples present in both the PPI network and test set were selected as a subset to further evaluate the ability of GTPLM-GO to extract information from the PPI network. The experimental results indicate that GTPLM-GO effectively utilizes PPI network data to improve protein function prediction performance.

Following the experimental setup of DeepGraphGO [32], we constructed two test subsets based on the presence or absence of protein samples in the PPI network, using the test set from the benchmark dataset. The two subsets are “STRING” and “HOMO”. STRING refers to proteins that are present in both test set and the PPI network; HOMO denotes proteins absent from the PPI network but homologous to those within PPI network; and NONE includes all other proteins. The number of STRING proteins in the MFO, BPO, and CCO are 286, 638, and 446, respectively, while the number of HOMO proteins are 132, 246, and 756. We then compared the performance of GTPLM-GO with advanced methods in STRING, and HOMO subsets. As shown in Table 2, GTPLM-GO outperforms other competing methods in 10 out of 12 metrics, with only a slight difference from DeepGraphGO in CCO of STRING (Fmax: −1.5%, AUPR: −0.2%). GTPLM-GO demonstrates superior performance in predicting protein functions in both STRING and HOMO. For instance, in the BP domain, GTPLM-GO (AUPR: 0.245) improves AUPR by 17.2% over DeepGraphGO (AUPR: 0.209) in STRING. Similarly, GTPLM-GO (AUPR: 0.192) outperforms DeepGraphGO (AUPR: 0.157) by 22.3% in HOMO. DeepGraphGO can be considered as the second best method in both STRING and HOMO subsets. Overall, network-based methods significantly outperform sequence-based methods, likely because they can utilize PPI information to predict protein functions, enabling the model to capture more functional associations among proteins.

### 2.5. Generalization on Specific Species

To evaluate the generalization capability of GTPLM-GO on species-specific proteins, we compared GTPLM-GO with advanced methods on the HUMAN and MOUSE test subsets. The benchmark dataset as well as the PPI network were constructed based on a multispecies strategy [32]. This strategy enabled the model to integrate multispecies PPI data during training. Then, the model predicts protein functions across various species. Therefore, to assess the generalization ability of GTPLM-GO on specific species, we first evaluated its performance on the species-specific test subsets of benchmark dataset. We use HUMAN and MOUSE proteins listed in Table 6 as instances, which is consistent with DeepGraphGO. Table 3 presents the comparison results of GTPLM-GO and advanced methods. GTPLM-GO achieved the best performance in 10 of the 12 metrics. Specifically, GTPLM-GO achieved optimal performances on the MOUSE test set in all metrics, while in the human test set, it only underperformed in MF. This suggests that GTPLM-GO trained using a multispecies strategy is more effective at extracting common features of proteins from multispecies PPI data.

Furthermore, we also investigated the ability of GTPLM-GO and DeepGraphGO to generalize to the HUMAN and MOUSE test set proteins when trained using only the target species proteins. This actually resulted in two variants, ${\mathrm{GTPLM-GO}}_{\mathrm{species}}$ and ${\mathrm{DeepGraphGO}}_{\mathrm{species}}$, respectively. Experimental results can be found in the Supplementary Materials (Table S2). Specifically, the performance of both variants generally declined when trained only on target species proteins. However, GTPLM-GO still outperforms DeepGraphGO on several domains. For example, in terms of AUPR, ${\mathrm{GTPLM-GO}}_{\mathrm{HUMAN}}$ and ${\mathrm{GTPLM-GO}}_{\mathrm{MOUSE}}$ achieve 0.764 and 0.641 on CCO, respectively, outperforming DeepGraphGO (0.642, 0.634). This further demonstrates the generalization of GTPLM-GO on specific species.

### 2.6. Ablation Studies

We performed ablation experiments to evaluate the contribution of the dual-branch Graph Transformer and protein language model. One component was removed at a time, and the model was retrained under the same experimental setup to assess the impact of each component on performance. Specifically, two variants were created: ${\mathrm{GTPLM-GO}}_{w/o\;\mathrm{ppi}}$, which retains only the functional semantic encoding of sequences generated by SeqVec to construct sequence-based embeddings of proteins, and ${\mathrm{GTPLM-GO}}_{w/o\;\mathrm{seq}}$, which retains only the dual-branch Graph Transformer to construct PPI-based embeddings. The detailed performance comparison of these variants with DeepGraphGO and GTPLM-GO is shown in Table 4. The results demonstrate that removing any component leads to a performance decline in GTPLM-GO, thus confirming that each component contributes positively to protein function prediction.

**Figure 2:**
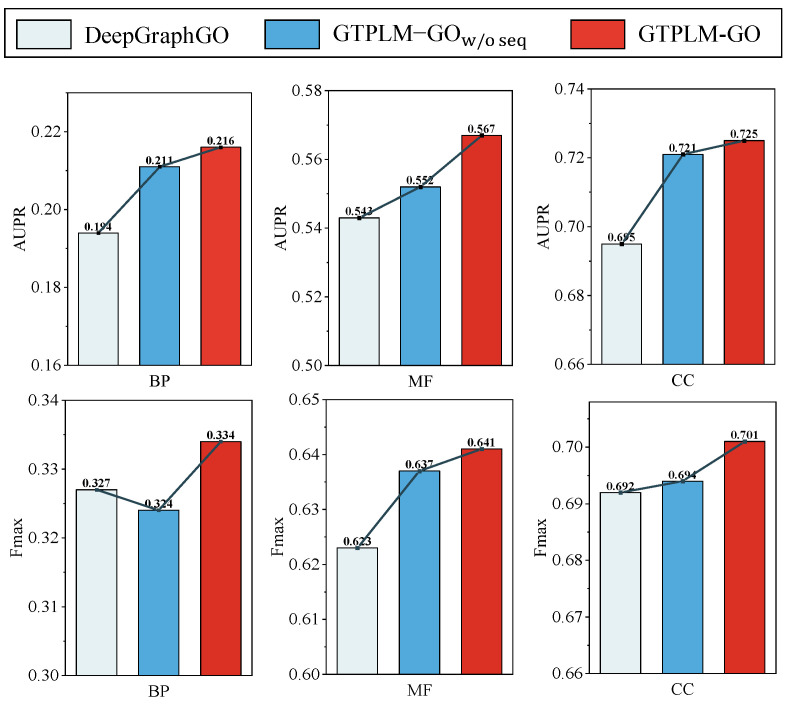
Performance comparison of GTPLM-GO, ${\mathrm{GTPLM-GO}}_{w/o\;\mathrm{seq}}$, and DeepGraphGO on the benchmark dataset.

Meanwhile, as shown in Table 4, the removal of the dual-branch Graph Transformer results in a significant performance drop (${\mathrm{GTPLM-GO}}_{w/o\;\mathrm{ppi}}$), emphasizing the crucial role of local–global PPI information in protein function prediction. Additionally, as shown in Figure 2, we validate the contribution of dual-branch Graph Transformer in extracting PPI network information by comparing the performance of ${\mathrm{GTPLM-GO}}_{w/o\;\mathrm{seq}}$, DeepGraphGO, and GTPLM-GO on the benchmark dataset. The results indicate that ${\mathrm{GTPLM-GO}}_{w/o\;\mathrm{seq}}$ performs the second-best in five out of six performance metrics, which suggests that the dual-branch Graph Transformer significantly outperforms DeepGraphGO in PPI network modeling. Simultaneously, the results also imply that the Graph Transformer has potential in the field of PPI network information mining and protein function prediction. Furthermore, GTPLM-GO achieves the best performance across all six metrics, highlighting that the integration of PPI network embedding with functional semantic coding of sequences constructed by the protein language model helps to enhance protein function prediction accuracy. It is also shown that the protein language model SeqVec is capable of extracting functional features that are not captured by the PPI network or InterPro features. We further refined the experimental setup for the ablation studies to assess the impact of removing the linear attention-based Transformer encoder and GCN on model performance. The specific experimental results can be found in the Supplementary Materials (Table S3).

**Figure 3:**
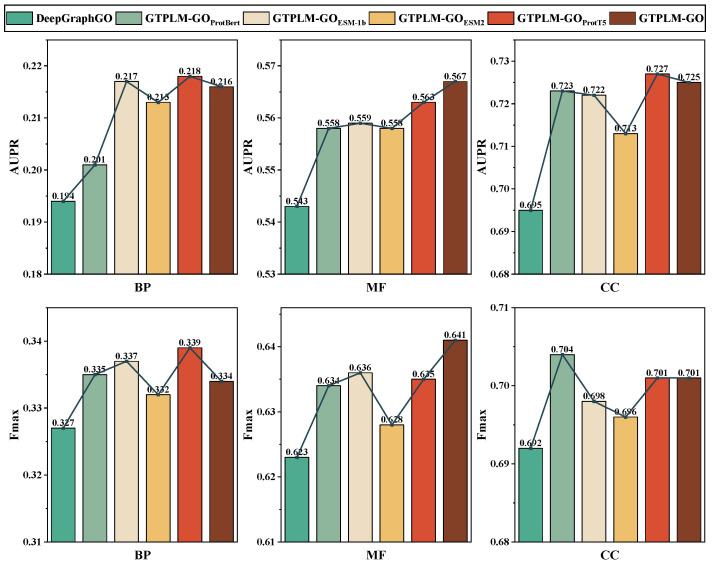
Performance comparison of GTPLM-GO variants using different protein language models on the benchmark dataset.

Subsequently, the impact of utilizing different protein language models (pLMs) on the prediction performance of GTPLM-GO was investigated. Specifically, we replaced the SeqVec model used in GTPLM-GO with four advanced pLMs: ProtBert [17], ESM-1b [18], ESM2 [50], and ProtT5 [17], resulting in four distinct variants. As shown in Table 5, SeqVec has the smallest number of parameters, which is 93 M. The parameter sizes of the ProtBert, ESM-1b, and ESM2 models we used are similar, at 420 M, 650 M, and 650 M, respectively. ProtT5 has the largest number of trainable parameters among the selected pLMs. These variants were retrained on the benchmark dataset, and their performance in protein function prediction was evaluated. As shown in Figure 3, GTPLM-GO achieved the best performance in MFO. ${\mathrm{GTPLM-GO}}_{\mathrm{ProtT}5}$ and ${\mathrm{GTPLM-GO}}_{\mathrm{ESM}-1b}$ attained the best and second-best performance in BPO, respectively. This may be due to the fact that the BPO task is more challenging on benchmark datasets. ESM-1b and ProtT5, with their larger parameter scales and more complex models, are better able to extract more information from the sequence, thereby improving performance on the BPO task. In CCO, ${\mathrm{GTPLM-GO}}_{\mathrm{ProtT}5}$ performed well, achieving the best Fmax and the second-best AUPR. ${\mathrm{GTPLM-GO}}_{\mathrm{ProtBert}}$ also performed well in CCO. Overall, both GTPLM-GO and its variants outperformed DeepGraphGO in all cases, suggesting that pLMs effectively capture knowledge relevant to functional prediction. Notably, although more advanced pLMs with more parameters, such as ESM-2 (650 M parameters) and ProtT5 (3B parameters), were used to construct variants of GTPLM-GO, no significant improvement was observed. However, this does not imply that larger-scale pLMs cannot yield potential performance gains. The limited performance differences suggest that the current GTPLM-GO architecture does not fully exploit the deep semantic information learned by pLMs. Therefore, future work will focus on optimizing the architectural design to better leverage the advantages of pLMs and enhance the accuracy of protein function prediction. Within the GTPLM-GO framework, the choice of different pLMs appears to have a relatively minor effect on performance. Given the relatively small number of trainable parameters in SeqVec (93M), we selected SeqVec to generate sequence feature semantic encodings in GTPLM-GO, prioritizing efficiency. Additionally, PPI data typically originate from prior knowledge, computational interaction predictions, and experimental data [51], often containing noise, which may be an important reason limiting the further improvement of the prediction accuracy of GTPLM-GO. We further performed hyperparameter studies, which can be found in the Supplementary Materials (Tables S4–S6, Figure S1).

## 3. Materials and Methods

### 3.1. Datasets

In this study, we utilize the benchmark dataset proposed by DeepGraphGO [32], which is collected following the standard Critical Assessment of Function Annotation (CAFA) protocol [7]. Specifically, the PPI network is constructed using a multispecies strategy, downloaded from STRING v11.0 [52], and protein sequences are obtained from UniProt [4]. GO terms are obtained from SwissProt [3], GOA [53], and GO [2]. Only experimental annotations (GO terms) with evidence codes: IDA, IPI, EXP, IGI, IMP, IEP, IC, or TA are retained. Protein function prediction can be regarded as a large-scale multilabel, multiclassification problem [12,32,44]. Three ontology domains, MFO, BPO, and CCO, in this dataset contain 6640, 21,288 and 2729 GO terms, respectively, corresponding to the number of labels to be predicted. The dataset is divided in accordance with DeepGraphGO and follows the CAFA protocol, where protein samples from multiple species are divided into train, valid, and test sets based on the timestamp of when the proteins were annotated. Table 6 shows the detailed statistics of the train, valid, and test sets, as well as the information of the subdatasets used in the extended experiments.

### 3.2. Methods

#### 3.2.1. Overview

**Figure 4:**
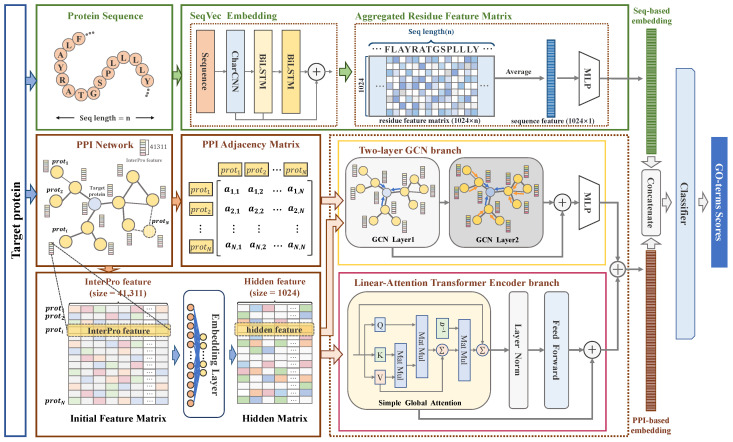
Overall architecture of GTPLM-GO: This model fuses sequence, local, and global PPI information to achieve protein function prediction. It develops a dual-branch Graph Transformer to collaboratively model both local and global information within PPI network. The Graph Transformer takes a PPI adjacency matrix and InterPro feature matrix as input. InterPro feature matrix is constructed by concatenating InterPro features [54] of proteins in the PPI network, while the PPI adjacency matrix encodes both direct and indirect protein interactions. The Graph Transformer extracts local PPI features through a two-layer GCN branch and captures global PPI features using a linear attention-based Transformer encoder. These features are then integrated via feature concatenation to generate PPI-based embeddings. Additionally, GTPLM-GO generates functional semantic representations of sequences using a protein language model, SeqVec [16] and constructs sequence-based embeddings through a multilayer perceptron (MLP). Finally, GTPLM-GO fuses sequence and PPI embeddings as inputs to the classifier to predict GO term scores.

As shown in Figure 4, GTPLM-GO develops a dual-branch Graph Transformer to enable the collaborative modeling of both local and global information within PPI networks. Subsequently, the PPI features extracted by the dual-branch Graph Transformer are combined with the functional semantic encoding of sequences generated by SeqVec. GTPLM-GO accepts two inputs: (1) PPI network consisting of N proteins (denoted as G). PPI network is represented by the weighted adjacency matrix $A\in R^{N\times N}$, where the element $a_{i,j}$ in the matrix denotes the confidence of an interaction between proteins ${prot}_{i}$ and ${prot}_{j}$ if the edge (*i*, *j*) exists, and 0 otherwise; (2) protein sequences.

For each protein ${prot}_{i}$ in the dataset, GTPLM-GO utilizes the dual-branch Graph Transformer to generate its PPI-based embedding. Specifically, a two-layer GCN branch is employed to extract features based on the local neighborhood and topological information of ${prot}_{i}$. Meanwhile, the global PPI functional features of ${prot}_{i}$ are captured using a linear attention-based Transformer encoder, with the resulting PPI-based embeddings derived through feature concatenation. Additionally, GTPLM-GO generates functional semantic encoding of a sequence via a protein language model and constructs the sequence-based embedding using an MLP. Finally, GTPLM-GO combines the PPI-based and sequence-based embeddings of ${prot}_{i}$ to obtain its final embedding and predicts the scores of ${prot}_{i}$ for each GO term through the GO term classifier.

#### 3.2.2. Linear Attention-Based Transformer Encoder for Extracting Global PPI Information

The linear attention-based Transformer encoder is a branch of the dual-branch Graph Transformer. It takes the PPI network (denoted as *G*) as input. The network *G* consists of *N* proteins. For each protein ${prot}_{i}$ in *G*, GTPLM-GO uses the InterPro feature $x_{i}\in R^{m}$ generated by InterProScan [55] as its initial feature, and *m* represents the feature dimension size. By concatenating the initial features of *N* protein nodes, we obtain the initial node feature matrix $X_{init}\in R^{N\times m}$. We then apply a node embedding layer to map the initial feature matrix $X_{init}$ to a low-dimensional hidden matrix $H^{\left(0\right)}\in R^{N\times d}$, which is computed as follows:

(4) $$H^{\left(0\right)}=\sigma \left(X_{init}W^{\left(0\right)}+b^{\left(0\right)}\right)$$

where $W^{\left(0\right)}\in R^{m\times d}$ and $b^{\left(0\right)}\in R^{d}$ represent the learnable weight matrix and bias, respectively, and σ is the nonlinear activation function.

Due to the constraints of the message-passing mechanism, GNNs face challenges in effectively extracting the global features of PPI networks through stacked graph network layers. SGFormer [40] is a simplified yet high-performance Graph Transformer. Inspired by the Simple Global Attention (SGA) mechanism of SGFormer [40], we propose that a single-layer attention network can effectively propagate protein feature information across the PPI network, enabling the modeling of potential functional associations between any pair of proteins within the PPI network. Therefore, we construct a Transformer encoder consisting of a single layer of the attention network based on the *SGA* mechanism proposed by SGFormer [40]. The SGA mechanism is defined as follows:

(5) $$Q=f_{Q}\left(H^{\left(0\right)}\right),K=f_{K}\left(H^{\left(0\right)}\right),V=f_{V}\left(H^{\left(0\right)}\right)$$

(6) $$D=\mathrm{diag}\left(1_{N}+\frac{1}{N}Q\left(K^{T}1_{N}\right)\right),\;\mathrm{SGA}=D^{-1}\left[V+\frac{1}{N}\tilde{Q}\left({\tilde{K}}^{T}V\right)\right]+H^{\left(0\right)}$$

where $f_{Q}$, $f_{K}$, $f_{V}$ denotes the linear layer used to construct *Q*, *K*, *V*, respectively, $1_{N}$ is the *N*-dimensional all-one vector, and the diag operator diagonalizes the *N*-dimensional vector into an N×N matrix. *Q* and *K* should be normalized using the Frobenius norm before the attention calculation. SGA eliminates the SoftMax operation, resulting in a linear time complexity of O(N), which enables scalability on large-scale PPI networks. The computational procedure of the linear attention-based Transformer encoder, based on the SGA mechanism, is as follows:

(7) $$H_{Trans}=\mathrm{MLP}\left(LN\left(\mathrm{SGA}\left(H^{\left(0\right)}\right)\right)\right)+H^{\left(0\right)}$$

where $H_{Trans}$ denotes the protein feature matrix constructed by the linear attention-based Transformer encoder branch, which enhances the ability of GTPLM-GO to model global information in PPI networks.

#### 3.2.3. Two-Layer GCN for Extracting Local PPI Information

Another component of the dual-branch Graph Transformer is the two-layer GCN. This branch builds upon the GCN proposed by [33], while preserving the ability to model direct(physical) interactions [52] in PPI networks. Inspired by DeepGraphGO [32], we integrate PPI edge weights and residual link [56] into the GCN, and the (l+1)−th layer is computed as follows:

(8) $$H_{\mathrm{GCN}}^{\left(l+1\right)}=\sigma \left({\tilde{D}}^{-\;\frac{1}{2}}\tilde{A}{\tilde{D}}^{-\;\frac{1}{2}}H_{GCN}^{\left(l\right)}W^{\left(l\right)}\right)+H_{GCN}^{\left(l\right)}$$

where $\tilde{A}=A+I_{N}$ represents the adjacency matrix of the network *G*, which includes the self-loop. Here, $I_{N}\in R^{N\times N}$ is the identity matrix, and $\tilde{D}\in R^{N\times N}$ denotes the degree matrix of $\tilde{A}$. Additionally, $W^{\left(l\right)}\in R^{d\times d}$ represents the learnable weights, σ is the nonlinear activation function, and $H_{GCN}^{\left(0\right)}=H^{\left(0\right)}$.

From the node perspective, the feature $h_{i}^{\left(l+1\right)}\in R^{d}$ of protein ${prot}_{i}$ at layer l+1 can be computed as

(9) $$h_{i}^{\left(l+1\right)}=h_{i}^{\left(l\right)}+\sigma \left(\sum_{j\in N(i)\cup \{i\}}{\mathrm{PPI}}_{i,j}\frac{1}{\sqrt{deg(i)}\sqrt{deg(j)}}W^{\left(l\right)}h_{j}^{\left(l\right)}\right)$$

where N(i) denotes the set of neighboring nodes of protein ${prot}_{i}$, and deg(i) and deg(j) represent the degrees of node ${prot}_{i}$ and its neighboring nodes, respectively. GCN aggregates the neighborhood features of the protein nodes, fully accounting for the direct interactions and local neighborhood information of the PPI network.

Finally, the dual-branch Graph Transformer combines the outputs of the linear attention-based Transformer encoder branch with the two-layer GCN branch to obtain the PPI-based embedding matrix $H_{PPI}\in R^{N\times 2d}$, as defined by the following equation:

(10) $$H_{PPI}=H_{GCN}^{\left(2\right)}\|H_{Trans}$$

The dual-branch graph Transformer of GTPLM-GO then achieves the collaborative modeling of both local and global information in PPI networks through a two-layer GCN and a linear attention-based Transformer encoder.

#### 3.2.4. Generating Functional Semantic Encoding Through Protein Language Model

The dual-branch Graph Transformer generates protein embeddings by considering the global perspective of biological networks. However, a protein’s sequence determines its structure and function, and these intrinsic features cannot be fully captured by the topological information of the PPI network alone. GTPLM-GO incorporates the protein language model SeqVec [16] to generate functional semantic encoding of sequences and then model the relationships between sequences and functions.

SeqVec comprises a CharCNN layer and two BiLSTM layers, enabling it to model sequences of arbitrary length. Firstly, CharCNN extracts local patterns from the sequence and generates a fixed-dimensional feature for each amino acid. Subsequently, the BiLSTM layers incorporate contextual information from the sequence. For a sequence ${prot}_{i}$ containing *n* amino acids, the feature ${SeqVec}_{j}$ of the *j*-th amino acid is computed as follows [16]:

(11) $${SeqVec}_{j}=h_{j}^{CharCNN}+h_{j}^{LSTM_{1}}+h_{j}^{LSTM_{2}}$$

where $h_{j}^{CharCNN}\in R^{1024}$ are the local features extracted by CharCNN, and $h_{j}^{LSTM_{1}}$ and $h_{j}^{LSTM_{2}}$ are the 1024-dimensional contextual features generated by the two BiLSTM layers, respectively. The 1024-dimensional feature of the *j*-th amino acid is derived by summing the features from the three SeqVec layers. The *n* residue-level features are then concatenated into a 1024×n dimensional feature matrix. Subsequently, the 1024-dimensional protein-level sequence features $h_{i}^{Seq}$ of protein ${prot}_{i}$ are derived by averaging these features. Finally, the sequence-based embedding of protein ${prot}_{i}$ is obtained through an MLP:

(12) $${emb}_{i}^{Seq}=f\left(W^{\left(1\right)}h_{i}^{Seq}+b^{\left(1\right)}\right)$$

where $W^{\left(1\right)}\in R^{d\times 1024}$ and $b^{\left(1\right)}\in R^{d}$ represent the learnable weight matrix and bias, respectively. In the implementation, SeqVec is pre-trained using UniRef50. To enhance the model’s running efficiency and facilitate its adaptation to large-scale datasets, we precompute the sequence encodings for the sequences of the proteins in the PPI network using SeqVec in an offline manner. This preprocessing step significantly reduces the computational overhead during model runtime. GTPLM-GO then combines sequence-based embeddings with PPI-based embeddings for protein function prediction.

It is worth noting that constructing models based on multibranch neural networks has become an effective approach to improve protein function prediction, such as DeepFMB [45], DeepFMB+ [45], SpatialPPIv2 [57], and Struct2GO [44]. These models perform tasks such as protein function prediction and PPI prediction by integrating various types of information, such as sequence, PPIs, orthology relations, and structural information. GTPLM-GO integrates PPI and sequence features to enhance predictive performance. GTPLM-GO utilizes a dual-branch Graph Transformer to achieve collaborative modeling of both local and global information in the PPI network, and extracts sequence features using a protein language model. A comparison between GTPLM-GO and the above methods can be found in the Supplementary Materials (Table S7).

#### 3.2.5. Protein Function Classifier Based on Sequence and Local–Global PPI Information

For protein ${prot}_{i}$, its embedding ${emb}_{i}$ is obtained by combining the PPI-based embedding ${emb}_{i}^{PPI}$ and the sequence-based embedding ${emb}_{i}^{Seq}$, as defined in the following equation:

(13) $${emb}_{i}={emb}_{i}^{PPI}\|emb_{i}^{Seq}$$

where ‖ denotes feature concatenation. Strategies such as weighted fusion and attention mechanisms may offer additional advantages. Concatenation was selected for its simplicity and effectiveness in our experiments. This strategy directly combines features from different sources while preserving the independence of features. Further explorations of integration strategies can be found in the Supplementary Materials (Table S8).

Subsequently, GTPLM-GO calculates the predicted score between proteins and GO terms using a GO term classifier, which consists of a fully connected layer, defined as follows:

(14) $${\left[{\hat{y}}_{i1},{\hat{y}}_{i2},...,{\hat{y}}_{iM}\right]}^{T}=f_{out}\left(emb_{i}\right)$$

where *M* denotes the number of GO terms, and ${\hat{y}}_{ij}\in R^{M\times 1}$ represents the confidence that protein ${prot}_{i}$ is predicted to belong to the *j*-th GO term. The classifier implements a mapping from protein embeddings to GO term scores.

GTPLM-GO employs binary cross-entropy loss as the loss function to optimize the model by minimizing the difference between true labels and predicted scores:

(15) $$BCELoss=-\frac{1}{BM}\sum_{i=1}^{B}\sum_{j=1}^{M}y_{ij}log\left({\hat{y}}_{ij}\right)+\left(1-y_{ij}\right)log\left(1-{\hat{y}}_{ij}\right)$$

where *B* denotes the number of proteins in one batch, and $y_{ij}\in \left\{0,1\right\}$ is the ground truth of protein ${prot}_{i}$.

## 4. Conclusions

In this paper, we propose GTPLM-GO, a method for protein function prediction by fusing sequence and local–global PPI information. GTPLM-GO achieves collaborative modeling of local and global information of the PPI network by utilizing a dual-branch Graph Transformer. Meanwhile, GTPLM-GO leverages the protein language model SeqVec to extract the functional semantic information of sequences and generates protein embeddings by combining them with PPI network features. GTPLM-GO then addresses the issue of insufficient information extraction in existing protein function prediction methods. Experimental results on datasets of different sizes demonstrate that GTPLM-GO outperforms advanced network-based and sequence-based methods in protein function prediction.

GTPLM-GO introduces a novel approach for extracting PPI network information, demonstrating the considerable potential of Graph Transformer in analyzing complex biological networks. While GTPLM-GO demonstrates excellent performance in protein function prediction, there is still room for further improvement. In the future, we aim to enhance both the applicability and prediction accuracy of GTPLM-GO while addressing issues within PPI networks, such as false positives and false negatives. Moreover, because a protein’s structure determines its function [58,59], highly accurate protein structure prediction techniques, such as AlphaFold2 [60], have driven the development of methods that utilize structural information to predict protein functions. A key focus of our future work will be integrating structural features into GTPLM-GO to further improve prediction accuracy. Based on the PDB files generated by AlphaFold2, we will construct residue contact maps and attempt to build protein structure encoders using GNNs or Graph Transformers to extract structural features. Additionally, we will explore ways to integrate structural features into GTPLM-GO, such as incorporating them as the initial features of protein nodes in the PPI network or as part of the initial features.

## Figures and Tables

**Table 1:** Performance comparison of GTPLM-GO and advanced methods on benchmark dataset *. **Bold** indicates the best performance, and underline indicates the second best.

Method	Fmax			AUPR		
	MFO	BPO	CCO	MFO	BPO	CCO
BLAST-KNN ^1^ [48]	0.590	0.274	0.650	0.455	0.113	0.570
LR-InterPro ^1^ [48]	0.617	0.278	0.661	0.530	0.144	0.672
Net-KNN ^1^ [49]	0.426	0.305	0.667	0.276	0.157	0.641
DeepGOCNN ^1^ [13]	0.434	0.248	0.632	0.306	0.101	0.573
DeepGOPlus ^2^ [13]	0.593	0.290	0.672	0.398	0.108	0.595
DeepGraphGO ^1^ [32]	0.623	0.327	0.692	0.543	0.194	0.695
GTPLM-GO ^2^	**0.641**	**0.334**	**0.701**	**0.567**	**0.216**	**0.725**

* Benchmark dataset refers to the dataset proposed by DeepGraphGO. ^1^ Single algorithm. ^2^ Composite algorithm.

**Table 2:** Performance comparison of GTPLM-GO with advanced methods on STRING and homologous proteins on the benchmark dataset *. **Bold** indicates the best performance, and underline indicates the second best.

Method	AUPR			Fmax		
	MFO	BPO	CCO	MFO	BPO	CCO
	STRING					
BLAST-KNN ^1^ [48]	0.466	0.122	0.438	0.608	0.291	0.570
LR-InterPro ^1^ [48]	0.562	0.162	0.598	0.630	0.293	0.627
Net-KNN ^1^ [49]	0.297	0.177	0.607	0.443	0.314	0.617
DeepGOCNN ^1^ [13]	0.173	0.036	0.136	0.432	0.258	0.588
DeepGOPlus ^2^ [13]	0.423	0.118	0.489	0.602	0.306	0.617
DeepGraphGO ^1^ [32]	0.582	0.209	**0.663**	0.642	0.348	**0.665**
GTPLM-GO ^2^	**0.607**	**0.245**	0.662	**0.653**	**0.350**	0.655
	HOMO					
BLAST-KNN ^1^ [48]	0.456	0.104	0.652	0.583	0.248	0.704
LR-InterPro ^1^ [48]	0.501	0.114	0.720	0.602	0.256	0.689
Net-KNN ^1^ [49]	0.253	0.128	0.675	0.422	0.300	0.709
DeepGOCNN ^1^ [13]	0.349	0.088	0.613	0.456	0.231	0.662
DeepGOPlus ^2^ [13]	0.438	0.100	0.656	0.582	0.257	0.710
DeepGraphGO ^1^ [32]	0.475	0.157	0.736	0.619	0.306	0.726
GTPLM-GO ^2^	**0.517**	**0.192**	**0.781**	**0.641**	**0.323**	**0.738**

* Benchmark dataset refers to the dataset proposed by DeepGraphGO. ^1^ Single algorithm. ^2^ Composite algorithm.

**Table 3:** Performance comparison of GTPLM-GO with advanced methods on HUMAN and MOUSE proteins on the benchmark dataset *. **Bold** indicates the best performance, and underline indicates the second best.

Method	AUPR			Fmax		
	MFO	BPO	CCO	MFO	BPO	CCO
	HUMAN					
BLAST-KNN ^1^ [48]	0.296	0.074	0.384	0.471	0.241	0.555
LR-InterPro ^1^ [48]	0.496	0.138	0.603	0.593	0.282	0.650
Net-KNN ^1^ [49]	0.358	0.143	0.620	0.485	0.261	0.615
DeepGOCNN ^1^ [13]	0.327	0.114	0.552	0.468	0.263	0.594
DeepGOPlus ^2^ [13]	0.246	0.088	0.479	0.501	0.277	0.625
DeepGraphGO ^1^ [32]	**0.520**	0.178	0.642	**0.633**	0.320	0.655
GTPLM-GO ^2^	0.471	**0.185**	**0.777**	0.588	**0.327**	**0.732**
	MOUSE					
BLAST-KNN ^1^ [48]	0.593	0.105	0.441	0.681	0.289	0.593
LR-InterPro ^1^ [48]	0.625	0.175	0.569	0.628	0.312	0.592
Net-KNN ^1^ [49]	0.319	0.167	0.569	0.420	0.302	0.588
DeepGOCNN ^1^ [13]	0.405	0.129	0.495	0.475	0.258	0.574
DeepGOPlus ^2^ [13]	0.550	0.132	0.488	0.634	0.306	0.598
DeepGraphGO ^1^ [32]	0.651	0.201	0.634	0.650	0.329	0.638
GTPLM-GO ^2^	**0.653**	**0.203**	**0.679**	**0.701**	**0.334**	**0.682**

* Benchmark dataset refers to the dataset proposed by DeepGraphGO. ^1^ Single algorithm. ^2^ Composite algorithm.

**Table 4:** Ablation study of dual-branch Graph Transformer and protein language model used by GTPLM-GO on the benchmark dataset. **Bold** indicates the best performance, and underline indicates the second best.

Method	Fmax			AUPR		
	MFO	BPO	CCO	MFO	BPO	CCO
${\mathrm{GTPLM-GO}}_{w/o\;\mathrm{ppi}}$	0.573	0.263	0.676	0.485	0.139	0.689
${\mathrm{GTPLM-GO}}_{w/o\;\mathrm{seq}}$	0.637	0.324	0.694	0.552	0.211	0.721
GTPLM-GO	**0.641**	**0.334**	**0.701**	**0.567**	**0.216**	**0.725**

**Table 5:** Detailed statistics of protein language models (pLMs) used in different GTPLM-GO variants.

Variant	Protein Language Model (pLM)	Parameters
GTPLM-GO	SeqVec	93 M
${\mathrm{GTPLM-GO}}_{\mathrm{ProtBert}}$	ProtBert	420 M
${\mathrm{GTPLM-GO}}_{\mathrm{ESM}-1b}$	esm1b_t33_650M_UR50S	650 M
${\mathrm{GTPLM-GO}}_{\mathrm{ESM}-2}$	esm2_t33_650M_UR50D	650 M
${\mathrm{GTPLM-GO}}_{\mathrm{ProtT}5}$	ProtT5-XL-UniRef50	3B

**Table 6:** Detailed statistics of benchmark dataset for three ontology domains: MFO, BPO, and CCO.

	Train			Valid			Test		
	MFO	BPO	CCO	MFO	BPO	CCO	MFO	BPO	CCO
All Data	51,549	85,104	76,098	490	1570	923	426	925	1224
Data used by GTPLM-GO	35,092	54,276	48,093	490	1570	923	426	925	1224
HUMAN (9606)	9208	12,095	18,842	86	138	137	41	87	767
MOUSE (10090)	6138	9927	8482	103	299	228	65	156	130

## Data Availability

The datasets and source codes (in Pytorch) of GTPLM-GO are available at https://github.com/gnahzt28/GTPLM-GO.

## Supplementary Materials

The following supporting information can be downloaded at https://www.mdpi.com/article/10.3390/ijms26094088/s1. References [13,32,33,41,44,45,48,49,54,57] are cited in the Supplementary Materials.

## References

[1] Eisenberg D., Marcotte E.M., Xenarios I., Yeates T.O. (2000). Protein function in the post-genomic era. Nature.

[2] Ashburner M., Ball C.A., Blake J.A., Botstein D., Butler H., Cherry J.M., Davis A.P., Dolinski K., Dwight S.S., Eppig J.T. (2000). Gene ontology: Tool for the unification of biology. Nat. Genet..

[3] Boutet E., Lieberherr D., Tognolli M., Schneider M., Bairoch A. (2007). UniProtKB/Swiss-Prot: The manually annotated section of the UniProt KnowledgeBase. Plant bioinformatics: Methods and protocols.

[4] The UniProt Consortium (2022). UniProt: The Universal Protein Knowledgebase in 2023. Nucleic Acids Res..

[5] Costanzo M., VanderSluis B., Koch E.N., Baryshnikova A., Pons C., Tan G., Wang W., Usaj M., Hanchard J., Lee S.D. (2016). A global genetic interaction network maps a wiring diagram of cellular function. Science.

[6] Radivojac P., Clark W.T., Oron T.R., Schnoes A.M., Wittkop T., Sokolov A., Graim K., Funk C., Verspoor K., Ben-Hur A. (2013). A large-scale evaluation of computational protein function prediction. Nat. Methods.

[7] Zhou N., Jiang Y., Bergquist T.R., Lee A.J., Kacsoh B.Z., Crocker A.W., Lewis K.A., Georghiou G., Nguyen H.N., Hamid M.N. (2019). The CAFA challenge reports improved protein function prediction and new functional annotations for hundreds of genes through experimental screens. Genome Biol..

[8] Altschul S.F., Gish W., Miller W., Myers E.W., Lipman D.J. (1990). Basic local alignment search tool. J. Mol. Biol..

[9] Altschul S.F., Madden T.L., Schäffer A.A., Zhang J., Zhang Z., Miller W., Lipman D.J. (1997). Gapped BLAST and PSI-BLAST: A new generation of protein database search programs. Nucleic Acids Res..

[10] Conesa A., Götz S., García-Gómez J.M., Terol J., Talón M., Robles M. (2005). Blast2GO: A universal tool for annotation, visualization and analysis in functional genomics research. Bioinformatics.

[11] Yuan Q., Xie J., Xie J., Zhao H., Yang Y. (2023). Fast and accurate protein function prediction from sequence through pretrained language model and homology-based label diffusion. Briefings Bioinform..

[12] Kulmanov M., Khan M.A., Hoehndorf R. (2018). DeepGO: Predicting protein functions from sequence and interactions using a deep ontology-aware classifier. Bioinformatics.

[13] Kulmanov M., Hoehndorf R. (2020). DeepGOPlus: Improved protein function prediction from sequence. Bioinformatics.

[14] Cao Y., Shen Y. (2021). TALE: Transformer-based protein function Annotation with joint sequence–Label Embedding. Bioinformatics.

[15] Vaswani A., Shazeer N., Parmar N., Uszkoreit J., Jones L., Gomez A.N., Kaiser Ł., Polosukhin I. Attention is all you need. Proceedings of the 31st International Conference on Neural Information Processing Systems.

[16] Heinzinger M., Elnaggar A., Wang Y., Dallago C., Nechaev D., Matthes F., Rost B. (2019). Modeling aspects of the language of life through transfer-learning protein sequences. BMC Bioinform..

[17] Elnaggar A., Heinzinger M., Dallago C., Rehawi G., Wang Y., Jones L., Gibbs T., Feher T., Angerer C., Steinegger M. (2021). Prottrans: Toward understanding the language of life through self-supervised learning. IEEE Trans. Pattern Anal. Mach. Intell..

[18] Rives A., Meier J., Sercu T., Goyal S., Lin Z., Liu J., Guo D., Ott M., Zitnick C.L., Ma J. (2021). Biological structure and function emerge from scaling unsupervised learning to 250 million protein sequences. Proc. Natl. Acad. Sci. USA.

[19] Peters M., Neumann M., Iyyer M., Gardner M., Zettlemoyer L. (2018). Deep Contextualized Word Representations. Proceedings of the 2018 Conference of the North American Chapter of the Association for Computational Linguistics: Human Language Technologies.

[20] Devlin J., Chang M.W., Lee K., Toutanova K. (2019). Bert: Pre-training of deep bidirectional transformers for language understanding. Proceedings of the 2019 Conference of the North American Chapter of the Association for Computational Linguistics: Human Language Technologies.

[21] Lin B., Luo X., Liu Y., Jin X. (2024). A comprehensive review and comparison of existing computational methods for protein function prediction. Briefings Bioinform..

[22] Szklarczyk D., Nastou K., Koutrouli M., Kirsch R., Mehryary F., Hachilif R., Hu D., Peluso M.E., Huang Q., Fang T. (2025). The STRING database in 2025: Protein networks with directionality of regulation. Nucleic Acids Res..

[23] Spirin V., Mirny L.A. (2003). Protein complexes and functional modules in molecular networks. Proc. Natl. Acad. Sci. USA.

[24] Zhang A. (2009). Protein Interaction Networks: Computational Analysis.

[25] Cui P., Wang X., Pei J., Zhu W. (2018). A survey on network embedding. IEEE Trans. Knowl. Data Eng..

[26] Perozzi B., Al-Rfou R., Skiena S. Deepwalk: Online learning of social representations. Proceedings of the 20th ACM SIGKDD International Conference on Knowledge Discovery and Data Mining.

[27] Mikolov T., Sutskever I., Chen K., Corrado G.S., Dean J. (2013). Distributed representations of words and phrases and their compositionality. Adv. Neural Inf. Process. Syst..

[28] Alshahrani M., Khan M.A., Maddouri O., Kinjo A.R., Queralt-Rosinach N., Hoehndorf R. (2017). Neuro-symbolic representation learning on biological knowledge graphs. Bioinformatics.

[29] Gligorijević V., Barot M., Bonneau R. (2018). deepNF: Deep network fusion for protein function prediction. Bioinformatics.

[30] Wu Z., Pan S., Chen F., Long G., Zhang C., Philip S.Y. (2020). A comprehensive survey on graph neural networks. IEEE Trans. Neural Netw. Learn. Syst..

[31] Yan K., Lv H., Guo Y., Peng W., Liu B. (2022). sAMPpred-GAT: Prediction of antimicrobial peptide by graph attention network and predicted peptide structure. Bioinformatics.

[32] You R., Yao S., Mamitsuka H., Zhu S. (2021). DeepGraphGO: Graph neural network for large-scale, multispecies protein function prediction. Bioinformatics.

[33] Kipf T.N., Welling M. (2016). Semi-supervised classification with graph convolutional networks. arXiv.

[34] Zhang X., Guo H., Zhang F., Wang X., Wu K., Qiu S., Liu B., Wang Y., Hu Y., Li J. (2023). HNetGO: Protein function prediction via heterogeneous network transformer. Briefings Bioinform..

[35] Li Q., Han Z., Wu X.M. (2018). Deeper insights into graph convolutional networks for semi-supervised learning. Proc. AAAI Conf. Artif. Intell..

[36] Chen D., Lin Y., Li W., Li P., Zhou J., Sun X. (2020). Measuring and relieving the over-smoothing problem for graph neural networks from the topological view. Proc. AAAI Conf. Artif. Intell..

[37] Ying C., Cai T., Luo S., Zheng S., Ke G., He D., Shen Y., Liu T.Y. (2021). Do transformers really perform badly for graph representation?. Adv. Neural Inf. Process. Syst..

[38] Wu Q., Zhao W., Li Z., Wipf D., Yan J. NodeFormer: A Scalable Graph Structure Learning Transformer for Node Classification. Proceedings of the Advances in Neural Information Processing Systems (NeurIPS).

[39] Wu Q., Yang C., Zhao W., He Y., Wipf D., Yan J. DIFFormer: Scalable (Graph) Transformers Induced by Energy Constrained Diffusion. Proceedings of the International Conference on Learning Representations (ICLR).

[40] Wu Q., Zhao W., Yang C., Zhang H., Nie F., Jiang H., Bian Y., Yan J. SGFormer: Simplifying and Empowering Transformers for Large-Graph Representations. Proceedings of the Advances in Neural Information Processing Systems (NeurIPS).

[41] Wu Z., Guo M., Jin X., Chen J., Liu B. (2023). CFAGO: Cross-fusion of network and attributes based on attention mechanism for protein function prediction. Bioinformatics.

[42] Lu J., Batra D., Parikh D., Lee S. (2019). ViLBERT: Pretraining Task-Agnostic Visiolinguistic Representations for Vision-and-Language Tasks. Proceedings of the Advances in Neural Information Processing Systems.

[43] Peng Z., Huang W., Gu S., Xie L., Wang Y., Jiao J., Ye Q. Conformer: Local features coupling global representations for visual recognition. Proceedings of the IEEE/CVF International Conference on Computer Vision.

[44] Jiao P., Wang B., Wang X., Liu B., Wang Y., Li J. (2023). Struct2GO: Protein function prediction based on graph pooling algorithm and AlphaFold2 structure information. Bioinformatics.

[45] Wang W., Shuai Y., Li Y., Zeng M., Li M. (2025). Enhancing Protein Function Prediction Through the Fusion of Multi-Type Biological Knowledge With Protein Language Model and Graph Neural Network. IEEE Trans. Comput. Biol. Bioinform..

[46] Kingma D.P., Ba J. (2014). Adam: A method for stochastic optimization. arXiv.

[47] Gligorijević V., Renfrew P.D., Kosciolek T., Leman J.K., Berenberg D., Vatanen T., Chandler C., Taylor B.C., Fisk I.M., Vlamakis H. (2021). Structure-based protein function prediction using graph convolutional networks. Nat. Commun..

[48] You R., Zhang Z., Xiong Y., Sun F., Mamitsuka H., Zhu S. (2018). GOLabeler: Improving sequence-based large-scale protein function prediction by learning to rank. Bioinformatics.

[49] You R., Yao S., Xiong Y., Huang X., Sun F., Mamitsuka H., Zhu S. (2019). NetGO: Improving large-scale protein function prediction with massive network information. Nucleic Acids Res..

[50] Verkuil R., Kabeli O., Du Y., Wicky B.I., Milles L.F., Dauparas J., Baker D., Ovchinnikov S., Sercu T., Rives A. (2022). Language models generalize beyond natural proteins. BioRxiv.

[51] Szklarczyk D., Gable A.L., Nastou K.C., Lyon D., Kirsch R., Pyysalo S., Doncheva N.T., Legeay M., Fang T., Bork P. (2020). The STRING database in 2021: Customizable protein–protein networks, and functional characterization of user-uploaded gene/measurement sets. Nucleic Acids Res..

[52] Szklarczyk D., Gable A.L., Lyon D., Junge A., Wyder S., Huerta-Cepas J., Simonovic M., Doncheva N.T., Morris J.H., Bork P. (2019). STRING v11: Protein–protein association networks with increased coverage, supporting functional discovery in genome-wide experimental datasets. Nucleic Acids Res..

[53] Huntley R.P., Sawford T., Mutowo-Meullenet P., Shypitsyna A., Bonilla C., Martin M.J., O’Donovan C. (2015). The GOA database: Gene ontology annotation updates for 2015. Nucleic Acids Res..

[54] Mitchell A.L., Attwood T.K., Babbitt P.C., Blum M., Bork P., Bridge A., Brown S.D., Chang H.Y., El-Gebali S., Fraser M.I. (2019). InterPro in 2019: Improving coverage, classification and access to protein sequence annotations. Nucleic Acids Res..

[55] Jones P., Binns D., Chang H.Y., Fraser M., Li W., McAnulla C., McWilliam H., Maslen J., Mitchell A., Nuka G. (2014). InterProScan 5: Genome-scale protein function classification. Bioinformatics.

[56] He K., Zhang X., Ren S., Sun J. Deep residual learning for image recognition. Proceedings of the IEEE Conference on Computer Vision and Pattern Recognition.

[57] Hu W., Ohue M. (2025). SpatialPPIv2: Enhancing protein–protein interaction prediction through graph neural networks with protein language models. Comput. Struct. Biotechnol. J..

[58] Holm L., Sander C. (1996). Mapping the protein universe. Science.

[59] Krissinel E. (2007). On the relationship between sequence and structure similarities in proteomics. Bioinformatics.

[60] Jumper J., Evans R., Pritzel A., Green T., Figurnov M., Ronneberger O., Tunyasuvunakool K., Bates R., Žídek A., Potapenko A. (2021). Highly accurate protein structure prediction with AlphaFold. Nature.

