# Supplementary material for: GTPLM-GO: Enhancing Protein Function Prediction Through Dual-Branch Graph Transformer and Protein Language Model Fusing Sequence and Local–Global PPI Information
Source: Int J Mol Sci. 2025 Apr 25;26(9):4088. doi: 10.3390/ijms26094088 (PMC12072039; doi:10.3390/ijms26094088)
Supplement: Supplementary file 1 [file ijms-26-04088-s001.zip › ijms-3559808-supplementary.pdf]

Supplemental Material

GTPLM-GO: Enhancing Protein Function Prediction through Dual-Branch Graph Transformer and Protein Language Model Fusing Sequence and Local-Global PPI Information

Haotian Zhang[1], Yundong Sun[1,2], Yansong Wang[1], Xiaoling Luo[3], Yumeng Liu[4], Bin Chen[1], Xiaopeng Jin[4 *], and Dongjie Zhu[1 *]

[1]School of Computer Science and Technology, Harbin Institute of Technology, Weihai 264209, China

[2]Department of Electronic Science and Technology, Harbin Institute of Technology, Harbin 150001, China

[3]College of Computer Science and Software Engineering, Shenzhen University, Shenzhen 518060, China

[4]College of Big Data and Internet, Shenzhen Technology University, Shenzhen 518118, China

Corresponding author: Xiaopeng Jin. (jinxiaopeng@sztu.edu.cn) Dongjie Zhu. (zhudongjie@hit.edu.cn)

A. Baselines on benchmark dataset

We compare the performance of GTPLM-GO with six baselines on the benchmark dataset introduced by DeepGraphGO, covering three ontology domains of Gene Ontology (GO). The results for these methods on the test set are directly obtained from DeepGraphGO. A description of these benchmark methods is provided below.

The six baselines consist of four sequence-based baselines: BLAST-KNN, LR-InterPro, DeepGOCNN, and DeepGOPlus. BLAST-KNN[48] operates under the assumption that proteins with similar sequences often share similar functions. LR-InterPro[48] is based on InterPro[54] features. These features are used as input to train logistic regression (LR) classifiers for each Gene Ontology (GO) term. DeepGOPlus[13] is a sequence-based method, and it combines DeepGOCNN[13] with a K-nearest neighbor algorithm called DiamondScore. DeepGOCNN uses a convolutional neural network to extract sequence features for function prediction, while protein similarity in DiamondScore is calculated using DIAMOND.

Two network-based baselines are Net-KNN and DeepGraphGO. The underlying theory of Net-KNN[49] is similar to that of BLAST-KNN; however, the similarity between proteins is determined by evaluating the edge weights in a protein-protein interaction (PPI) network constructed from the STRING database. DeepGraphGO[32] uses InterPro features as input and learns the features of proteins from their local

neighborhoods in the PPI network using two layers of Graph Convolutional Networks (GCN)[33]. It then outputs the predicted scores between each protein and each Gene Ontology (GO) term through a fully connected layer. DeepGraphGO is one of the most advanced network-based methods.

We can also divide baselines into single algorithms and composite algorithms. Composite algorithms can be regarded as consisting of multiple individual algorithms or components. GTPLM-GO can be regarded as a composite algorithm, consisting of a dual-branch Graph Transformer and a protein language model. BLAST-KNN and LR-InterPro are components of GOLabeler and can be regarded as single algorithms. Net-KNN is a component of NetGO and can also be considered a single algorithm. DeepGOPlus is composed of DiamondScore and DeepGOCNN, so DeepGOCNN is a single algorithm, while DeepGOPlus is a composite algorithm. DeepGraphGO is composed of a two-layer GCN, so it is regarded as a single algorithm.

B. CFAGO Dataset

To further evaluate the performance of GTPLM-GO, we introduce a benchmark dataset proposed by CFAGO (CFAGO dataset). CFAGO[41] is a protein function prediction model that utilizes an encoder-decoder architecture based on the pre-training and fine-tuning paradigm. The model employs the Transformer encoder architecture to learn more effective protein representations from multiple sources, including protein-protein interactions (PPIs) from a single species network and protein sequences, thereby enhancing protein function prediction performance.

Table S1: Detailed statistics of CFAGO dataset for three ontology domains MFO, BPO and CCO.

Species	Train			Valid			Test		
	MFO	BPO	CCO	MFO	BPO	CCO	MFO	BPO	CCO
HUMAN	2747	3197	5263	503	304	577	719	182	119
MOUSE	1185	2714	4014	232	336	694	126	155	147

CFAGO dataset comprises two single-species subsets: HUMAN dataset and MOUSE dataset. All configurations in our study align with those of CFAGO, utilizing PPI data and protein sequences from the STRING (v11.5) database, and protein subcellular location and structural domain data from the UniProt database. The dataset is divided into training, validation, and test sets according to the CAFA specification. CFAGO dataset excludes GO terms for proteins annotated in more than 5% of the single-species PPI network. In HUMAN subset, the MFO, BPO, and CCO categories contain only 38, 45, and 35 GO terms, respectively, while the MOUSE subset contains 17, 42, and 37 GO terms. Detailed statistics for the CFAGO dataset are provided in Table S1. For CFAGO dataset, we use DeepGraphGO and CFAGO as baselines. We used the PPI network structural features, domain and subcellular location features in CFAGO dataset as initial features of the PPI Network. The evaluation metrics used in this paper are m-AUPR and Fmax. Experimental results for DeepGraphGO and

CFAGO are directly taken from the original CFAGO paper.

C. Generalization on specific species

Table S2: Performance comparison of GTPLM-GO and DeepGraphGO in generalizing to HUMAN and MOUSE test subset when trained using only target species proteins. GTPLM-GO$_{species}$ and DeepGraphGO$_{species}$ denote the model trained and evaluated using only specific species. GTPLM-GO and DeepGraphGO denote the model trained using the full benchmark dataset. **Bold** indicates the best performance, while <u>underline</u> indicates the second best.

Methods	Fmax			AUPR		
	MFO	BPO	CCO	MFO	BPO	CCO
			HUMAN			
DeepGraphGO$_{HUMAN}$	**0.636**	0.299	0.629	**0.530**	0.163	0.601
DeepGraphGO	<u>0.633</u>	<u>0.320</u>	0.655	<u>0.520</u>	<u>0.178</u>	0.642
GTPLM-GO$_{HUMAN}$	0.618	0.318	<u>0.731</u>	0.449	0.177	<u>0.764</u>
GTPLM-GO	0.588	**0.327**	**0.732**	0.471	**0.185**	**0.777**
			MOUSE			
DeepGraphGO$_{MOUSE}$	0.559	0.309	0.602	0.499	0.183	0.584
DeepGraphGO	0.650	<u>0.329</u>	0.638	<u>0.651</u>	<u>0.201</u>	0.634
GTPLM-GO$_{MOUSE}$	<u>0.657</u>	0.306	<u>0.666</u>	0.613	0.181	<u>0.641</u>
GTPLM-GO	**0.701**	**0.334**	**0.682**	**0.653**	**0.203**	**0.679**

D. Ablation study

Table S3: Results on ablation study. We additionally introduce two variants: GTPLM-GO$_{w/o\ GNN}$ denotes removing the two-layer GNN of GTPLM-GO, and GTPLM-GO$_{w/o\ Trans}$ denotes removing the linear-attention-based Transformer encoder of GTPLM-GO. **Bold** indicates the best performance, while <u>underline</u> indicates the second best.

Methods	Fmax			AUPR		
	MFO	BPO	CCO	MFO	BPO	CCO
GTPLM-GO$_{w/o\ ppi}$	0.573	0.263	0.676	0.485	0.139	0.689
GTPLM-GO$_{w/o\ seq}$	<u>0.637</u>	0.324	<u>0.694</u>	<u>0.552</u>	<u>0.211</u>	<u>0.721</u>
GTPLM-GO$_{w/o\ GNN}$	0.617	0.286	0.687	0.547	0.149	0.704
GTPLM-GO$_{w/o\ Trans}$	0.611	<u>0.330</u>	0.692	0.548	0.200	0.713
GTPLM-GO	**0.641**	**0.334**	**0.701**	**0.567**	**0.216**	**0.725**

E. Hyperparameter studies

We analyze the effect of the number of attention layers and the number of attention heads of the linear attention-based Transformer encoder on the performance of GTPLM-GO within benchmark dataset.

Table S4: Evaluate the effect of different linear attention heads on the performance of GTPLM-GO within the range {1, ... ,4,6,8}. Bold indicates the best performance.

Methods	Fmax			AUPR		
	MFO	BPO	CCO	MFO	BPO	CCO
$\text{GTPLM-GO}_{\text{head}=1}$	**0.641**	**0.334**	**0.701**	**0.567**	**0.216**	**0.725**
$\text{GTPLM-GO}_{\text{head}=2}$	0.626	0.331	0.695	0.550	0.206	0.716
$\text{GTPLM-GO}_{\text{head}=3}$	0.625	0.330	0.691	0.553	0.208	0.709
$\text{GTPLM-GO}_{\text{head}=4}$	0.604	0.329	0.697	0.541	0.210	0.714
$\text{GTPLM-GO}_{\text{head}=6}$	0.629	0.328	0.694	0.563	0.209	0.710
$\text{GTPLM-GO}_{\text{head}=8}$	0.622	0.328	0.695	0.545	0.209	0.720

Table S5: Evaluate the effect of different linear attention layers on the performance of GTPLM-GO within the range {1, ... ,4,6,8}. Bold indicates the best performance.

Methods	Fmax			AUPR		
	MFO	BPO	CCO	MFO	BPO	CCO
$\text{GTPLM-GO}_{\text{layer}=1}$	**0.641**	**0.334**	**0.701**	**0.567**	**0.216**	**0.725**
$\text{GTPLM-GO}_{\text{layer}=2}$	0.621	0.330	0.692	0.551	0.207	0.719
$\text{GTPLM-GO}_{\text{layer}=3}$	0.613	0.333	0.691	0.543	0.211	0.709
$\text{GTPLM-GO}_{\text{layer}=4}$	0.601	0.331	0.687	0.521	0.212	0.697
$\text{GTPLM-GO}_{\text{layer}=6}$	0.608	0.330	0.689	0.536	0.211	0.708
$\text{GTPLM-GO}_{\text{layer}=8}$	0.601	0.323	0.690	0.524	0.201	0.699

Figure S1: Evaluate the effect of different numbers of linear attention heads on the performance of GTPLM-GO at layer=1 (A). Evaluate the effect of different numbers of linear attention layers on the performance of GTPLM-GO at head=1 (B).

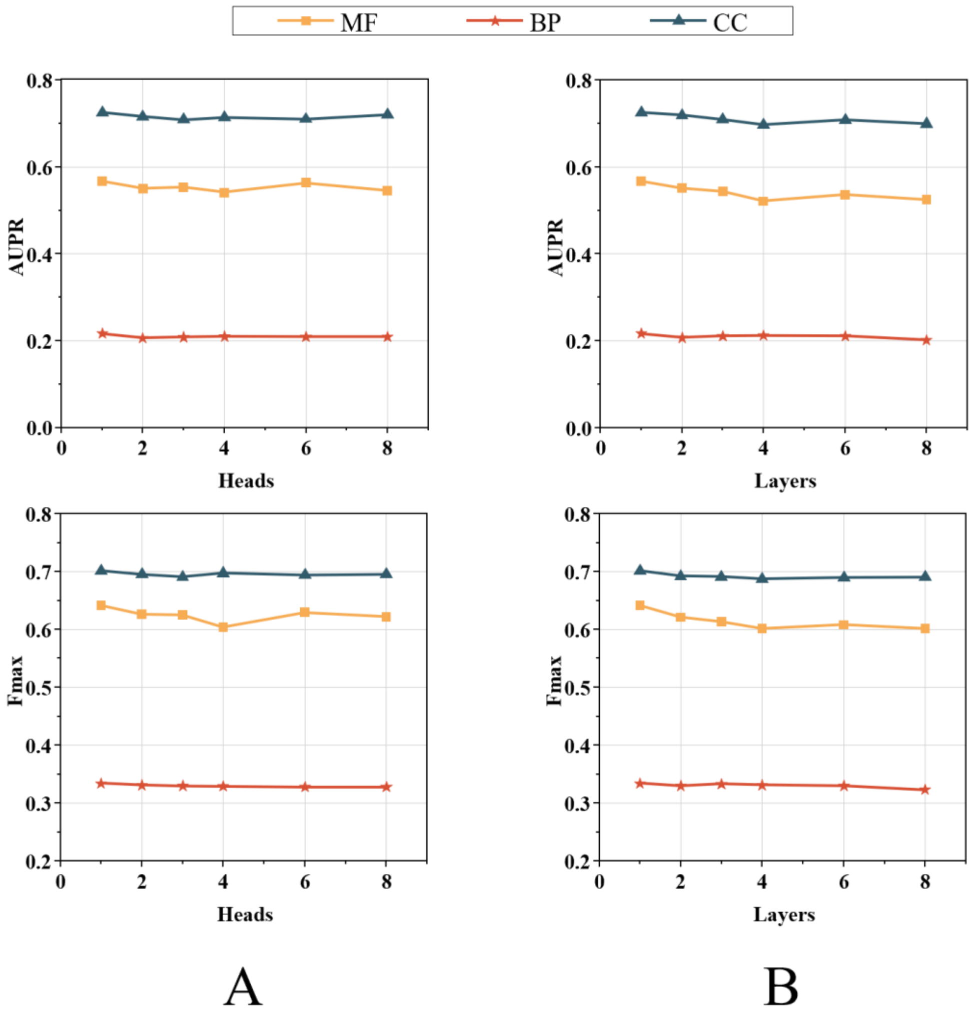

Table S6: In the case of layer=2 and layer=4 (multiple linear attention layers), evaluate the effect of different linear attention heads on the performance of GTPLM-GO within the range {1,... ,4,6,8}. Bold indicates the best performance. Bold indicates the best performance.

Methods	Fmax			AUPR		
	MFO	BPO	CCO	MFO	BPO	CCO
GTPLM-GO$_{layer=1,head=1}$	**0.641**	**0.334**	**0.701**	**0.567**	**0.216**	**0.725**
GTPLM-GO$_{layer=2,head=2}$	0.609	0.329	0.694	0.538	0.209	0.711
GTPLM-GO$_{layer=2,head=3}$	0.622	0.332	0.697	0.562	0.212	0.722
GTPLM-GO$_{layer=2,head=4}$	0.615	0.332	0.696	0.543	0.215	0.723
GTPLM-GO$_{layer=2,head=6}$	0.612	0.329	0.698	0.544	0.207	0.722
GTPLM-GO$_{layer=2,head=8}$	0.621	**0.334**	0.700	0.548	0.212	0.723
GTPLM-GO$_{layer=4,head=2}$	0.601	0.326	0.695	0.532	0.206	0.721
GTPLM-GO$_{layer=4,head=3}$	0.600	0.327	0.685	0.536	0.199	0.696
GTPLM-GO$_{layer=4,head=4}$	0.605	0.332	0.692	0.534	0.209	0.706
GTPLM-GO$_{layer=4,head=6}$	0.611	0.332	0.692	0.544	0.211	0.707
GTPLM-GO$_{layer=4,head=8}$	0.602	0.331	0.693	0.546	0.206	0.712

F. Model Comparison

Table S7: Comparison of models based on multi-branch neural networks.

Method	Input	Model architecture
DeepFMB[45]	Sequences, PPIs, Orthology relations	GCN, Protein Language Model
DeepFMB+[45]	Sequences, PPIs, Orthology relations	GCN, Protein Language Model, BlastKNN
SpatialPPIv2[57]	Sequences, Structural information	GAT, Protein Language Model
Struct2GO[44]	Sequences, Structural information	GCN, Protein Language Model
GTPLM-GO	Sequences, PPIs	Protein Language Model, Dual-branch Graph Transformer

DeepFMB[45] integrates various forms of biological knowledge, including protein sequences, PPIs, and orthology relations, to improve protein function prediction performance. DeepFMB+[45] further enhances predictive performance by combining the results of DeepFMB with sequence similarity-based methods BlastKNN. Both DeepFMB and DeepFMB+ demonstrate the positive impact of integrating multiple types of biological knowledge for protein function prediction. SpatialPPIv2[57] captures the residue relationships between two proteins through GAT and extracts residue sequence information using a protein language model, achieving high-performance PPI prediction. Struct2GO[44] uses GCN and a protein language model to extract structural and sequence features for protein function prediction.

Compared to the strategy of integrating multiple forms of biological knowledge in DeepFMB and DeepFMB+, GTPLM-GO achieves protein function prediction by integrating PPI features and sequence features. GTPLM-GO focuses on improving protein function prediction performance by enhancing information extraction from PPI Networks. Specifically, it uses a dual-branch Graph Transformer to improve the model's ability to collaboratively model local and global information from the PPI Network, and then extracts sequence features using a protein language model. By integrating sequence and PPI features, GTPLM-GO enhances protein function prediction.

G. Integration strategies

We further investigated the impact of the weighted fusion strategy on the performance of GTPLM-GO on benchmark dataset. We aligned the dimensions of sequence features and PPI features, and then performed weighted fusion using five different $weight$ parameters ($weight = \{0.2, 0.4, 0.5, 0.6, 0.8\}$):

$$emb_i = weight * emb_i^{PPI} + (1 - weight) * emb_i^{Seq}$$

Through the weighted fusion strategy, we evaluated the effect of feature fusion with different weights on the performance of the GTPLM-GO model. Although the current weighted fusion strategy has not improved performance, we plan to further explore more effective weighted fusion strategies. For example, we aim to optimize the weighted fusion effect by dynamically adjusting the weights. In future work, we will continue to explore different feature fusion strategies and evaluate their potential applications in GTPLM-GO.

Table S8: Evaluate the effect of different weight on the performance of GTPLM-GO within the range {0.2, 0.4, 0.5, 0.6, 0.8}. Bold indicates the best performance.

Methods	Fmax			AUPR		
	MFO	BPO	CCO	MFO	BPO	CCO
GTPLM-GO$_{weight=0.2}$	0.569	0.293	**0.686**	0.494	0.165	**0.700**
GTPLM-GO$_{weight=0.4}$	0.598	0.298	0.684	**0.524**	0.171	0.683
GTPLM-GO$_{weight=0.5}$	**0.604**	**0.300**	0.680	0.517	**0.172**	0.676
GTPLM-GO$_{weight=0.6}$	0.603	0.289	0.680	0.513	0.160	0.675
GTPLM-GO$_{weight=0.8}$	0.602	0.287	0.673	0.507	0.157	0.664
